# ORAL HEALTH, PERCEIVED STRESS, AND SUBJECTIVE MEMORY COMPLAINTS ACCORDING TO DIABETES STATUS

**DOI:** 10.1093/geroni/igae098.4248

**Published:** 2024-12-31

**Authors:** Min Jung Kim

**Affiliations:** Gachon University, Incheon, Republic of Korea

## Abstract

Oral health status has been linked to cognitive impairment. People with diabetes often experience more oral health issues and higher emotional stress than those without, that may increase the risk of cognitive impairment. This study explored the relationship between oral health, perceived stress, and subjective memory complaints (SMC), and whether these relationships differed by diabetes status, using data from the 2021 Korean National Health and Nutrition Examination Survey. Perceived oral health, stress levels, and SMC were assessed via questionnaires, and the number of natural teeth was counted during exams. Tobit regression models were used, with subgroup analyses by diabetes status. Among 1,444 adults, after controlling for demographic factors, perceived oral health (r = 0.16, p <.05) and stress levels (r = 0.16, p <.05) significantly increased SMC in healthy adults (n = 504). However, only stress levels increased SMC in those with prediabetes (n = 602, r = 0.29, p <.001) and diabetes (n = 260, r = 0.19, p <.05). The number of natural teeth did not affect SMC in any group. These findings suggest perceived oral health may indicate early cognitive decline in healthy adults, while psychological status is a more critical factor in those with prediabetes or diabetes. These results suggest that perceived oral health may be an early indicator of cognitive decline in healthy adults, while psychological health plays a more dominant role in cognitive decline in adults with prediabetes or diabetes. Tailored interventions to prevent cognitive decline should consider diabetes status.

